# “Tell me what you suggest, and let’s do that, doctor”: Patient deliberation time during informal decision-making in clinical trials

**DOI:** 10.1371/journal.pone.0211338

**Published:** 2019-01-29

**Authors:** Haruka Nakada, Sachie Yoshida, Kaori Muto

**Affiliations:** Department of Public Policy, Human Genome Center, The Institute of Medical Science, University of Tokyo, Tokyo, Japan; University of Sydney, AUSTRALIA

## Abstract

Informed consent is an essential part of an ethical clinical trial; to this end, researchers have developed several interventions to promote participants’ full understanding of trials and thereby improve the consent process. However, few empirical studies have examined how patients make the decision of whether to give consent. The objective of this study, therefore, is to analyze patients’ decision-making process when participating in clinical trials. We conduct an internet survey (n = 2,045) and interview data analysis (n = 40) with patients and categorize respondents into three types of participants: active, passive, and non-participation. Our results show that patients often make informal and quick decisions before medical staff provide them with relevant information during the informed consent process. For example, 55.9% of patients received initial information on clinical trials from an online article or web advertising, and 54.5% consulted no one about whether to participate in the clinical trial before making a decision. Only 20.7% of respondents subjectively spent time making the decision whether to participate; 43.0% of patients who said that they “spent time” coming to a decision took four or more days to reach a decision, while 8.3% of people who “did not spend time” making a decision took this among of time. Based on these results, we were able to break patients’ decision-making process into four steps: first contact, informal decision making, relevant information, and formal decision making. Our results show that patients are most likely to make a decision based on the first information they receive on the clinical trial, whatever the source. To this end, having a list of questions for potential participants to ask researchers would be useful in helping better collecting information of clinical trials. In addition, research teams should give patients more than four days to decide between providing them with relevant information and obtaining written consent, even if the patient seems to make a quick decision.

## Introduction

Clinical trials have social value, producing scientific knowledge that can lead to improvements in public health and medical care. Participation of patients are necessary for clinical trials, and as such must be protected based on ethical principles. The Declaration of Helsinki dictates current guidance and regulations for patient treatment [1]: patients must be given the opportunity to provide informed consent based on a “respect for persons.” This means that in order to give consent, individuals must be accurately informed of the purpose, methods, risks, benefits, and alternatives to the research; understand this information and its bearing on their own clinical situation; and make a voluntary and uncoerced decision on whether to participates [2]. To this end, investigators must provide potential participants with all relevant information before they enroll in a clinical trial.

Current guidelines, regulations, and practices tend to emphasize the ways to provide information to potential participants or list the information that should be provided to potential participants, instead of referring to the decision-making process; for example, the U.S. Common Rule addresses eight basic elements of information that should be provided to each participant [3]. Likewise, the several studies that have attempted to better understand participants’ decision-making regarding clinical trials focus on the content and structure of information (e.g., enhancing the information provided or changing the presentation format) rather than the decision-making process. And yet, the value of audio-visual interventions in helping enhance the informed consent process for potential clinical trial participants remains largely unclear [4] and there is insufficient evidence of whether decision aids are more effective in decision-making than standard information [5]. In addition, current informed consent documents do not encourage good-quality decision-making among clinical trial participants [6–7].

One way to improve existing studies is to take patients’ experiences and voices into account when improving the informed consent process. Though Locock [8] examines 42 patients’ overall experience in clinical trials, few empirical studies have focused solely on patients’ decision-making process. Existing studies tend to focus on patients’ experiences in a specific situation. For example, Kohara and Inoue [9] examine the decision-making process of 25 cancer patients considering participation in Phase 1 clinical trials. These patients had to address end-of-life issues: whether to gamble on the possibility of a successful clinical trial or to focus on palliative care. Kohara and Inoue’s findings detailed the cancer patients' decision-making process in Phase 1 clinical trials and the relevant issues; however, these findings cannot explain common issues in other types of clinical trials.

Another lack in previous research is that the definition of “good decision-making” in clinical trials has been controversial, especially in clinical practice, as the idea of shared decision-making has emerged. Shared decision-making has been defined as “an approach where clinicians and patients share the best available evidence when faced with the task of making decisions, and where patients are supported to consider options, to achieve informed preferences” [10]. Elwyn [11] proposes a distinction between the act of decision-making and the process of coming to that decision—what he calls determination and deliberation. He argues that the quality of a patient’s decision-making can be measured by evaluating this deliberation phase rather than the determination itself or the outcome of the determination. In other words, good deliberation is the basis of good determination.

However, Elwyn’s work focuses on understanding patient behavior when interacting with their primary physicians inside a hospital. This model must be adapted to fully understand the decision-making process during a clinical trial, since such a decision includes factors both inside and outside a hospital. When considering how medical experts in a hospital can promote informed consent and decision-making in a clinical trial setting, therefore, we have to divide patients’ decision-making process—or Elwyn’s deliberation stage—into private and public deliberation.

Our previous research shows that patients often make an “informal decision” regarding their participating in a clinical trial even before they receive detailed information from medical experts. All the patients in our study remained true to their primary “informal decision” when signing formal consent forms [12]. This suggests that understanding how patients’ deliberation before meeting medical experts affects their informed consent is an important factor in understanding the informed consent process during trial participation [13].

As described above, while studies have examined how to develop better communication tools to improve participant recruitment and support both patients’ initial decision and ongoing participation [14], it remains unclear exactly how a patient decides to participate in clinical trials before visiting hospitals. The objective of this study, therefore, is to analyze patients’ informal decision-making process when deciding whether to participate in clinical trials and identify the essential points during this process.

## Materials and methods

This study consists of two datasets: quantitative data and interview data. First, we conducted an internet survey that collected quantitative data regarding patients’ decision-making during clinical trials. Based on the results of this, we extracted relevant interview data from the existing database to analyze individuals’ backgrounds and decision-making process.

### Internet survey

We sent an online survey to 2,688 adult patients (> 20 years old) who enrolled in clinical trials conducted from 2013 to 2016 in Japan. We defined a “clinical trial” as an industry-funded drug trial to collect data for regulatory approval and excluded academically funded studies. These patients were extracted from the survey panel of INTAGE Incorporated. The data collection period was March 7–9, 2017. The questionnaire included items on basic characteristics, patients’ knowledge of clinical trials, and their decision-making process regarding their participation. We analyzed the data from the respondents who gave informed consent.

Our survey was out of the scope of Japan’s Ethical Guidelines for Medical and Health Research Involving Human Subjects, and there are no national guidelines in Japan for social and behavioral research. Therefore, our study was carried out in accordance with the Ethical Principles for Sociological Research of the Japan Sociological Society, which do not require ethical reviews. After being informed about the purposes of this study and the general subject matter of the questions, participants agreed to participate in the survey. Completing the entire questionnaire was considered participant consent.

### Interview analysis

We collected interview data that had been stored in the database managed by DIPEx Japan, which collaborates with academic researchers to provide free, reliable information about health issues by sharing people's real-life experiences. The database contained interview data of 40 Japanese patients, who had some relevant experiences with clinical trials. We then coded the sentences in these 40 patients’ transcripts using qualitative data analysis software (MAXQDA 10) to extract and categorize the factors relevant to the informed consent process. We developed the coding framework based on the qualitative data analysis introduced by DIPEx, which employed constant comparison of transcripts, analyzed deviant cases, and identified both anticipated and emergent themes [15]. Two authors (HN and SY) independently analyzed the coding reports, and we discussed differences in interpretation to resolve any disagreements.

## Results

### Patient characteristics

#### Internet survey

The response rate of the online survey was 76.1% (n = 2,045). Of the 2,045 respondents, 1,473 gave informed consent and received experimental drugs. The average age was 55.0 (Table 1).

**Table 1:** Characteristics of online survey.

N = 1,473		n	%
Gender	Male	1,061	72.0
	Female	412	28.0
Age	20–29	51	3.5
	30–39	136	9.2
	40–49	274	18.6
	50–59	423	28.7
	60–69	417	28.3
	70–79	172	11.7
Education	Junior high school	15	1.0
	Senior high school	319	21.7
	College	231	15.7
	University	808	54.9
	Graduate school	93	6.3
	Other, N/A	7	0.4
Household income	< $40,000	383	26.0
	$40,000–$60,000	293	19.9
	$60,000–$100,000	439	29.8
	> $100,000	231	15.7
	N/A	127	8.6
Disease	Hypertension	419	28.4
	Diabetes	235	16.0
	Dyslipidemia	183	12.4
	Allergies	166	11.3
	Heart disease	58	3.9
	Depression	57	3.9
	Cancer	34	2.3
Experience with clinical trials	Completed	1,254	85.1
	Dropout	202	13.7
	Withdrawal	17	1.2
Current health condition	After discharge	39	2.6
	Regular office visit	783	53.2
	Office visit as needed	250	17.0
	Home healthcare	10	0.7
	Other, N/A	391	26.5

Clinical trial literacy was significantly higher among the patients in 60–79 age group, who had fewer correct answers to the question regarding “randomization” (50.0%) than others (e.g., the percentage of correct answers in the question regarding “placebo” was 87.1% in 60–79 age group).

#### Interview analysis

Participants included 12 men and 28 women (including one romantic couple) aged between 27 and 81. Several patients had taken part in more than one trial, sometimes for different conditions (Table 2). All the data, including conditions, experiences, and types of intervention, were self-reported. Some patients experienced a clinical trial not directly relevant to their condition, such as a clinical trial for a drug to prevent the side effects of another drug they were taking for their condition or a clinical trial for an anesthetic drug they received during a surgery to treat their condition.

**Table 2:** Characteristics of interviewees.

n = 40 (ages 27–81)		n	%
Gender	Male	14	35
	Female	26	65
Disease	Cancer	13	32.5
	Not cancer	27	67.5
Experience with clinical trials	Completed	23	57.5
	Dropped out for medical reasons	11	27.5
	Wish to participate but not enrolled	5	12.5
	Requested to participate but not enrolled	1	2.5
	Other	2	5.0
Position	Patient	37	92.5
	Family member	3	7.5
Type of clinical trial	Drug	34	82.5
	Device	3	7.5
	Others	4	10

### Internet survey

#### Patients’ deliberation time

Many patients received their initial information on clinical trials from online article/web advertising (51.8%) or medical staff, including their primary physicians (15.2%). Younger patients tended to receive initial information from TV (12.8% among 20–39 age group, 2.4% among those 60–79) and family members/friends (9.6% among 20–39 age group, 3.1% among 60–79 age group). Elderly patients, on the other hand, were more likely to receive information from online articles/web advertising (41.2% among 20–39 age group, 58.1% among 60–79 age group). The percentage of people who received their first information from medical staff was similar in each age group: 18.2%, 13.3%, and 16.5% among those ages 20–39, 40–59, and 60–79, respectively.

**Fig 1:**
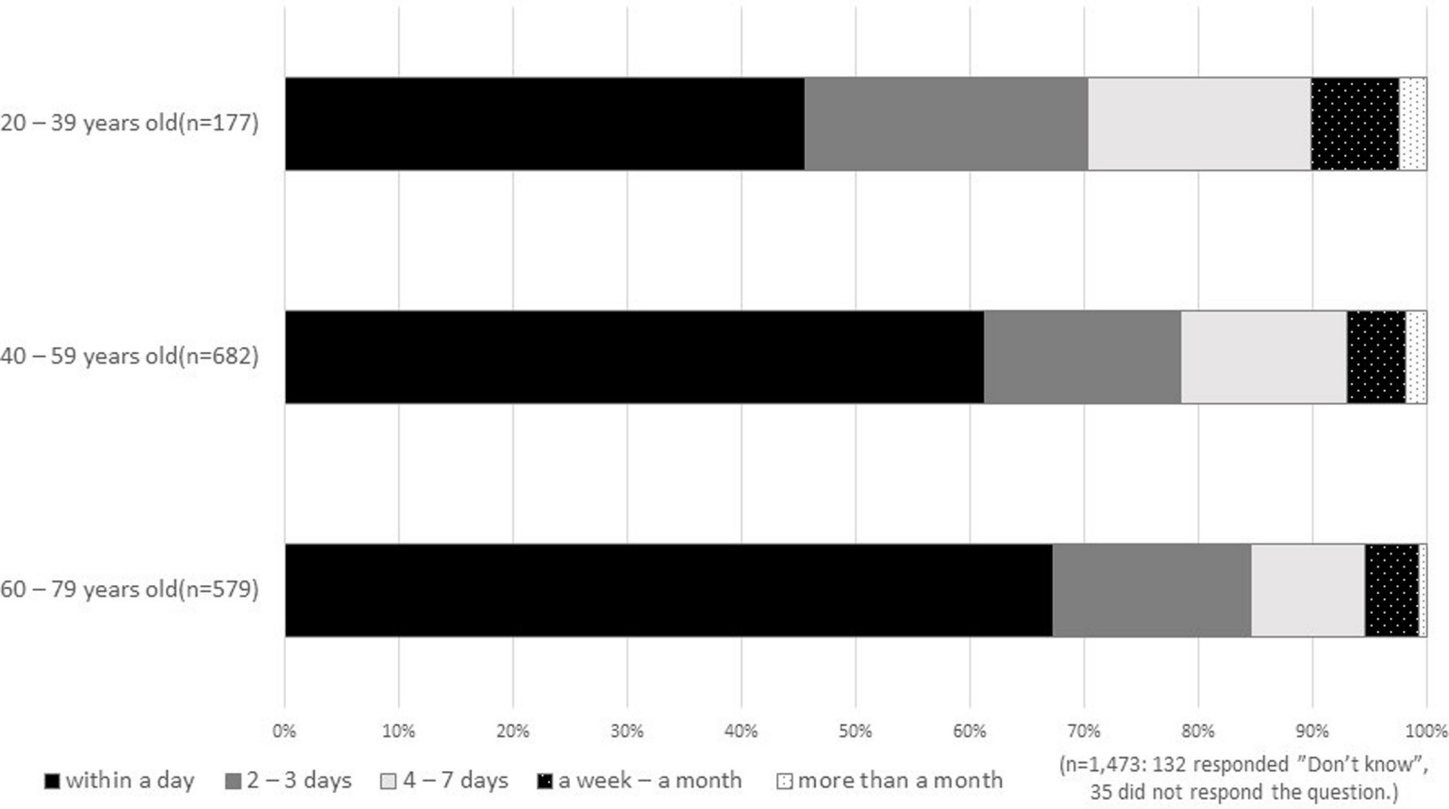
Survey respondents’ time spent in decision-making.

Patients did not spend much time deliberating on their participation in a clinical trial. Among the 1,473 patients who gave informed consent and received experimental drugs, 20.7% spent subjectively “some or much time” deciding whether to participate. Elderly patients spent significantly less time deciding than younger ones (Fig 1): 40.7% of 20–39 year olds and 61.7% of 60–79 year olds made a decision within a day.

**Fig 2:**
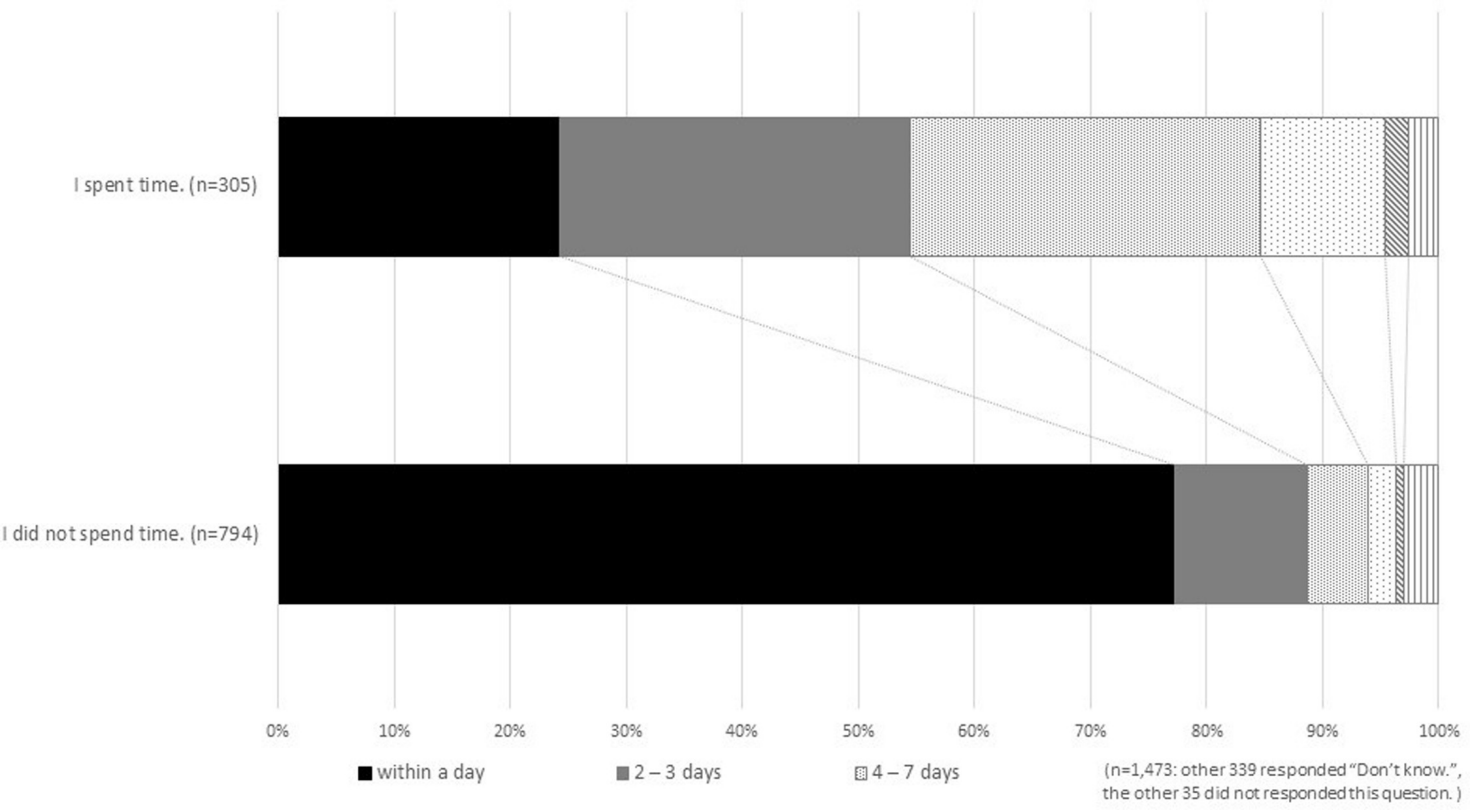
Survey respondents’ time spent deliberating in relation to their subjective description of decision-making time.

Patients varied on the days they spent deliberating and their subjective feelings regarding whether they spent enough time to make a decision. Fig 2 shows the range of deliberation time taken by patients before they made their decision. Overall, 43.0% of the patients who said that they “spent time” coming to a decision took four or more days to reach a decision, while 8.3% of people who “did not spend time” making a decision took this among of time (Fig 2).

#### Patient consultation with others

A majority of patients did not discuss their participation in the clinical trial with others (e.g., family members, primary physicians). Among the 1,473 patients who did participate in a trial, 54.5% talked with no one before their decision-making. Meanwhile, 83.4% of patients took the consent form home and read it again alone.

### Interview analysis

The database managed by DIPEx Japan had interviews on a variety of topics regarding clinical trials extracted from patients’ voices: basic information, patients’ information sources, the reason they decided to participate, their experiences giving informed consent, messages from patients to medical experts, etc. We extracted relevant interviews from the dataset, including those that addressed patients’ information sources, the reason(s) they decided (not) to participate, and their experiences giving informed consent. Our data collection focused on their decision-making process.

#### Decision-making process

Among the 40 patients included in our analysis, three patients wondered whether they would participate in a clinical trial. Most patients who decided to participate in a clinical trial didn’t change their mind during the trial period, with two exceptions. Ct39 withdrew from a trial because she moved and ct40 withdrew because of financial reasons. Others had to quit the trial due to their medical condition, not because they changed their mind. One patient, Ct16, said that her doctor ended her participation without any explanation—the interviewers could not figure out why based on her story.

Our analysis showed that patients tended to come to decisions in one of three ways: active participation, passive participation, or non-participation. Active participants included patients who applied to a clinical trial by themselves, those who sought out information on a clinical trial relevant to their condition, and those who requested detailed information on a clinical trial without any recommendation from others. These were often patients who first received information on a clinical trial from their primary physicians. For example, one of the interviewees was a man with lymphocytic leukemia (ct19); he had an ongoing constructive relationship with his physician and often went to the hospital for follow-up visits. During one visit, while talking about treatment strategies, his physician mentioned a clinical trial comparing the efficacy of existing drugs to that of combination drug therapy. The physician told him it was up to patients’ decision and suggested he go home to talk with his family members whether he participated or not. But the man replied:

“No, no, my wife will say, ‘You should decide yourself,’ and my children will say, ‘It’s your own business.’ So, I said to the doctor, ‘Let’s decide now. Tell me what you suggest, and let’s do that.’ My doctor told me he wanted to try the combination drug therapy. So I said, ‘Let’s do it. I’ll be ok, things usually work out for me.’ And we decided to try the combination therapy.”

The man elicited a definite opinion from the physician, then decided to adopt his idea.

Passive participants included patients who accepted based on others’ suggestion without considering it for themselves. This included patients who applied to a clinical trial in response to a recruiting advertisement and patients who complied with the suggestion of their primary physicians without asking any questions. For example, ct11 was a woman with chronic myeloid leukemia who agreed to participate in a clinical trial after her physician suggested it. She had just begun treatment but was suffering from severe side effects. While she waited to be admitted into the hospital for treatment of these side effects, her physician suggested a clinical trial. In the interview, she said, “My doctor said, ‘If I were you, I would surely participate.’ So, I decided to participate in the clinical trial immediately.”

The final group, non-participants, included those who could not or decided not to participate in a clinical trial. The reason varied: some didn’t meet the inclusion criteria, others were concerned about stopping their current medication, and some doubted the methods of a clinical trial. Non-participating patients lost interest in clinical trials altogether. For example, ct20, a woman with fibromyalgia, had two non-participation experiences and said she felt rejected when she had dropped out her trial. Another woman, ct30, who had a spinal cord injury, described patients’ attitudes toward clinical trials, saying that patients who were not interested in clinical trials should be accepted as they are.

Overall, regardless of the decision-making process, the patients in the interviews we analyzed had few experiences discussing their participation with medical staff or actively asking questions regarding clinical trials until they had already come to a decision. Instead, they were more likely to consult people around them, including family members, or look online for more information.

Patients of all decision-making types also tended to come to a quick informal decision and stick with this decision throughout the trial period. Ct23, for example, was invited to participate in a clinical trial on diabetes by her primary physician (passive participation). She felt uncomfortable and painful drawing her own blood every day during the trial period; despite this, however, she strongly wished to accomplish her hospital visits till the end as she was instructed by her clinical trial staff. She talked she never wished to withdraw and stood by her decision to participate. Her participation type, in other words, played a minor role in whether she stood by her choice to participate.

## Discussion

**Fig 3:**
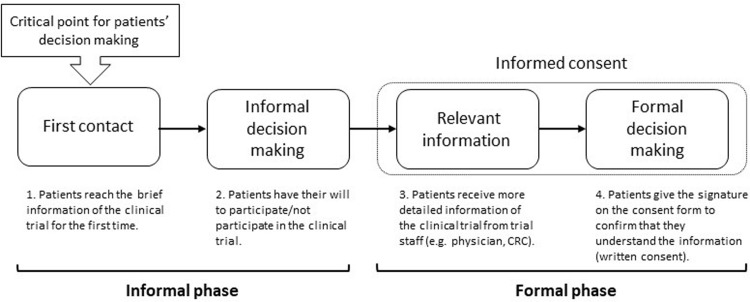
Patients’ decision-making process.

We determined patients’ detailed decision-making process by compiling the quantitative and qualitative data from both parts of our study. We determined that patients went through four decision-making steps when deciding whether to participate in a clinical trial (Fig 3). These took places in two phases: the “informal” and “formal” phase. Each phase includes two steps: first contact and informal decision-making in the informal phase and relevant information and formal decision-making in the formal phase. Patients proceed to formal decision-making based on the relevant information they receive; however, as our previous study demonstrate, most patients had made an informal decision prior to the formal informed consent process [12]. Understanding this informal phase is a vital to analyzing patients’ decision-making.

During the first step, “first contact,” patients encounter information on the clinical trial for the first time, whether from their primary physicians, web advertisements, blogs or hospital advertisements. This is the most crucial point in patients’ decision-making because by the first impression of this information will orient patients towards a clinical trial. Patients, especially elderly patients, make their decision regarding clinical trials quickly without consulting anybody.

Our results also show that although the internet is an important source of information for patients, elderly patients are less likely to rely on the internet for health-related information, a result that corresponds to those of previous studies [16]. Instead, they may have trust in their physicians and leave medical decisions to them. Previous studies [17] have found that patients use the internet to have an active role in their own health decisions; however, even these patients have high trust in their physicians and value consultations with them. Our interview survey, however, found that patients did not collect relevant information for decision-making through communication with medical experts. Rather, few patients communicated with medical experts or asked questions, even during the formal informed consent process [18]: previous studies have found that 27% of patients in clinical trials for the treatment of chronic medical conditions did not ask any questions [18–19]. Even if patients have questions, they tend to ask no more than four questions [19]. In Japan and other countries, patients tend to hesitate or feel uncomfortable to have a conversation with experts regarding clinical trials, to varying degrees.

Half of the patients in our interview survey had “first contact” through medical staff, most of who were their primary physicians. Because in this case patients may hesitate to reject their primary physician’s offer to participate, physicians should inform patients that they have a variety of resources regarding clinical trials. In addition, primary physicians should clarify that they do not have enough detailed information to allow patients to make informed decisions: investigators or clinical research coordinators are more knowledgeable than primary physicians. However, patients may feel uncomfortable meeting with relevant experts without any instruction. To this end, providing patients with a list of helpful questions to ask may benefit them and allow them to make better use of their information resources, including investigators or clinical research coordinators. The U.S. National Institutes of Health (NIH), which has published a similar list of questions to ask, also tells potential participants, “Write down any questions you might have and bring your list with you when you first meet with the research team” [20–21]. This suggests to potential participants that the first meeting with the research team is important. In Japan, some of the institutions have published a Q&A list for patients who are invited to participate in clinical trials.

Another way to do this is to develop e-consent platforms that help patients and the research team address any relevant questions. Researchers have worked on using multimedia tools to make informed consent more engaging [22]. Rowbotham et al. [23], for example, develop an interactive consent system on an iPad to help patients ask appropriate questions to the on-site research team. They find that patients spent an average of 13.2 minutes reviewing paper consent forms, while they spent an average of 22.7 minutes on the iPad system, which included an introductory video, consent form, and interactive quiz with 12 multiple-choice comprehension and recall questions. Patients using the iPad also scored significantly higher on the quiz, meaning that they better recalled the contents of the consent form than the patients using the paper form.

Whatever the method, research teams must confirm the patients’ deliberate decision on whether to participate in a clinical trial. The research team should not provide a patient with relevant information on the clinical trial and obtain written consent on the same day. To this end, each patient should be guaranteed with an adequate amount of time to deliberate, even though more than half of patients make a decision within one day. Without this time a patient’s informal quick decision will be accepted as their voluntary will. Our internet-based survey showed that patients felt like they had spent an adequate amount of time, of at least four days, to make a decision. Thus, medical staff should give patients more than four days between explaining the trial and obtaining written consent. If patients make the quick decision to not participate in a clinical trial, the research team should give them a deadline by which to change their minds, rather than just offering them the opportunity to provide consent again.

Finally, our results highlighted that patients’ diligent attitudes to accomplish their roles as trial participants in spite of knowing right to withdraw, less benefits for their health, pain and uncomfortableness. We couldn’t conclude by our study whether this attitude was formed independently of the impact of “first contact” or quick decision, however, medical experts should communicate with participants during the trial period to relax them not to endure too much.

Our study has some limitations. Primarily, it remains unclear whether participants who make a quick decision not to participate in a clinical trial will feel the same throughout the trial period. In the current study, we focus on participants’ initial decision to participate rather than to abstain. This was done for two reasons: participants who consent without understanding potential risks may suffer more risks and it is more difficult to collect data on patients who decided not to participate in a clinical trial. In addition, we cannot ignore recall bias in the internet survey. There was a time lag between their actual experiences and the survey, though we recruited participants of clinical trials that were as recent as possible. The next step would be an embedded survey regarding their thoughts in clinical trials.

Our results may reflect Japan's own experience of having achieved universal health coverage (UHC) in 1961 and the reciprocal but somewhat paternal relationship between physicians and patients. We need further empirical studies to compare our results with those in other countries with different healthcare systems and patient-physician relationships.

## Conclusion

This study determines that patients tend to make quick and informal decision regarding whether to participate in clinical trials. While they come to this decision in different ways, they maintain this initial decision throughout the trial period. In order to ensure the ethical consent of participants, therefore, the research team needs to wait more than four days after providing potential participants with relevant information before obtaining the written consent.

## Supporting information

S1 Internet survey questionnaire in English (DOCX)

S1 Internet survey questionnaire in Japanese (DOCX)
