# Supplementary material for: “Tell me what you suggest, and let’s do that, doctor”: Patient deliberation time during informal decision-making in clinical trials
Source: PLoS One. 2019 Jan 29;14(1):e0211338. doi: 10.1371/journal.pone.0211338 (PMC6350979; doi:10.1371/journal.pone.0211338)
Supplement: S1 Internet survey questionnaire in English — (DOCX) [file pone.0211338.s001.docx]

**A survey on experiences participating in clinical trials related to the development of new pharmaceuticals/medical devices and related attitudes**

This survey asks about your thoughts and experiences regarding your assistance in the past with clinical trials related to the development of new pharmaceuticals and medical devices, as well as your attitudes regarding these trials. The results will be used to create systems for enhancing the development of new pharmaceuticals and medical devices. If you have trouble answering any of the survey questions, you may skip them. In addition, participation is voluntary.

**This survey is administered to patients who have participated in clinical trials within the past three years**, and is being conducted by the University of Tokyo, with financial assistance from the Japan Agency for Medical Research and Development.

In this survey, your name and personal information will not be revealed. Further, the results will not be used for any purpose other than research. Thank you for your assistance.

If you have read and understood the above and are willing to provide assistance with the survey, please proceed to answer the questions.

**When a new pharmaceutical is developed, the study conducted to confirm its safety and efficacy is referred to as the “clinical trial” or the “chiken.” For instance, a study may be conducted to determine the efficacy of taking a pharmaceutical, equipping a device, or undergoing an operation or examination that is in development.**

**First, please choose the answer you believe is correct in response to the questions below concerning clinical trials/“chiken”.**

1-1. Clinical trials/“chiken” are conducted for the purpose of the future advancement of medicine. (Choose only one answer)

(a) Correct

(b) Incorrect

(c) Not sure

1-2. Some clinical trials/“chiken” involve taking a safe placebo that does not contain active pharmaceutical components for purposes of comparison with a pharmaceutical under development. (Choose only one answer)

(a) Correct

(b) Incorrect

(c) Not sure

1-3. In some clinical trials/“chiken”, patients may not be able to decide whether to undergo an existing treatment or a treatment under development. (Choose only one answer)

(a) Correct

(b) Incorrect

(c) Not sure

1-4. It may not be possible for a person to participate in a clinical study/clinical trial, depending on the severity of their illness. (Choose only one answer)

(a) Correct

(b) Incorrect

(c) Not sure

1-5. A clinical trial/“chiken” leads to future treatments and standard treatments being established. (Choose only one answer)

(a) Correct

(b) Incorrect

(c) Not sure

1-6. Patients are not responsible for the costs of treatments and pharmaceuticals that are part of a clinical trial/“chiken”. (Choose only one answer)

(a) Correct

(b) Incorrect

(c) Not sure

1-7. Patients may be paid for participating in a clinical study/clinical trial in order to reduce the patients’ burden of expenses. (Choose only one answer)

(a) Correct

(b) Incorrect

(c) Not sure

1-8. When participating in a clinical study/clinical trial, throughout a certain time period, it is necessary to regularly receive treatment/be hospitalized. (Choose only one answer)

(a) Correct

(b) Incorrect

(c) Not sure

1-9. Japanese patients may be unable to use a pharmaceutical that is standard in other countries because it has not been approved in Japan.

(a) Correct

(b) Incorrect

(c) Not sure

1-10. Japanese patients may be unable to undergo treatment using a certain medical device or examination when that medical device or examination is standard in other countries, but not yet approved in Japan.

(a) Correct

(b) Incorrect

(c) Not sure

**The following questions will ask about your experiences with clinical trials.**

*** Clinical trials include the following:**

**- Studies where patients take a new pharmaceutical whose efficacy is being investigated**

**- Studies where patients wear a device that is not approved for sale in Japan whose efficacy is being investigated**

2. When did you assist, offer to assist, or receive a request to participate in the clinical trial? Please answer using a Western date or Japanese date. If you have multiple experiences, please answer regarding the most recent one.

Showa year ( ), Heisei year ( ), or Western calendar year ( ), approximately

3. Please indicate where you first heard of the clinical trial.

(a) A program or advertisement on television

(b) An article or advertisement in a newspaper or magazine

(c) An article or advertisement on the Internet

(d) An invitation by hospital staff

(e) A poster or pamphlet at the hospital

(f) Family, friends, or acquaintances

(g) Information received from a patient group

(h) Information received at a presentation or seminar for the general public

(i) Other (fill in your answer: )

4. Did you receive detailed explanatory documents regarding the clinical trial (for instance, a request to participate in a study)?

(a) Yes **→ go to 5**

(b) No **→ answer complete**

(c) Don’t remember **→ answer complete**

**These questions are for respondents who answered “Yes” to question 4, “Did you receive detailed explanatory documents regarding the clinical trial (for instance, a request to participate in a study)?”.**

5. Did a doctor, nurse, or other person explain the documents to you?

(a) Yes, mainly a doctor

(b) Yes, mainly a nurse

(c) Yes, mainly the clinical research coordinator

(d) No **→ go to 16**

(e) Do not remember **→ go to 16**

5. Was the explanation you were given easy to understand?

(a) Extremely easy to understand

(b) Fairly easy to understand

(c) Did not understand very well

(d) Did not understand at all

(e) Do not remember

8. Were you given an explanation of the negative physical effects, side effects, and so on that could result from participating in the clinical trial?

(a) Yes

(b) No

(c) Do not remember

9. Was it explained to you that you were free to decide to quit the clinical trial even if you had initially agreed to participate?

(a) Yes

(b) No

(c) Do not remember

10. Did you take the documents home and read them?

(a) Yes, I read them alone

(b) Yes, I read them with family or friends

(c) No

(d) Do not remember

11. Do you still have these documents currently?

(a) I have kept them

(b) I disposed of them

(c) I do not know where I put them

(d) Do not remember

12. Did you speak with someone or ask for someone’s advice regarding whether to participate in the clinical trial? (multiple answers)

(a) Hospital staff, such as a doctor or nurse

(b) Family members

(c) Friends or acquaintances

(d) An acquaintance with the same illness

(e) Did not ask for anyone’s advice

(f) Other (fill in your answer: )

13. Did you take your time and consider whether to participate in the clinical trial? Additionally, please state the reason why.

(a) I took an extremely long time and thought it over well [fill in your answer: ]

(b) I took some time and thought it over [fill in your answer: ]

(c) I did not take much time to think it over [fill in your answer: ]

(d) I did not take any time to think it over at all [fill in your answer: ]

(e) Do not remember

14. Why did you decide to participate in the clinical trial? (multiple answers)

(a) Because I felt I could contribute to the advancement of medical care

(b) Because I felt I could contribute to the development of treatment methods for my illness

(c) Because I wanted to show gratitude for the medical care I have received

(d) Because I thought my illness might improve

(e) Because I thought I could receive new treatments (including new pharmaceuticals)

(f) Because I had no choice but to participate in the clinical trial to cure my illness

(g) I was satisfied with a trial doctor’s explanation

(h) Because I was compensated for participation in the clinical trial

(i) Because I couldn’t refuse the doctor’s request

(j) Other (fill in your answer: )

15. When you decided to participate in the clinical trial/“chiken”, did you have the option of choosing a different treatment method? (multiple answers)

(a) There were no other treatment methods, so I chose the clinical trial

(b) It would have been possible to choose a traditional treatment method, but I chose the clinical trial

(c) I do not know if there were any other treatment methods

**Currently, some people feel that it is necessary to improve communication with patients after the clinical trial’s conclusion. These questions ask about your opinion regarding this.**

16. At this point, how do you feel about participating in the clinical trial?

(a) Very satisfied

(b) Satisfied

(c) OK

(d) Dissatisfied

(e) Very dissatisfied

17. Please state the reason.

[Fill in your answer: ]

18. Would you like to participate in a clinical trial in the future?

(a) I definitely would

(b) I suppose so

(c) I am not sure

(d) Not really

(e) Definitely not

19. Do you know if the pharmaceutical, medical device, and so on of the clinical trial you participated in was sold following the completion of the clinical trial?

(a) I know **→ go to 20**

(d) I do not know **→ go to 21**

20. How did you find out?

(a) Heard from a doctor

(b) Heard from the clinical research coordinator

(c) Looked it up on my own

(d) Other (fill in your answer: )

21. Do you want to know whether the pharmaceutical, medical device, and so on of the clinical trial you participated in was sold following the completion of the clinical trial?

(a) Yes, I do

(b) No, I do not

(c) Not sure

22. How would you like to be informed? Keep in mind that at present, the drug manufacturer does not have your personal information. (multiple answer allowed).

(a) Would like to receive documents in the mail from the hospital

(b) Would like the hospital to tell me by phone

(c) Would like for posters to be hung or pamphlets to be left in the hospital

(d) Would like the hospital to provide the information by email or a social network

(e) Not sure

**In some clinical trials, patients assist by taking a safe placebo that does not contain pharmaceutical components for a certain period of time in order to compare its efficacy with that of the pharmaceutical under development. In this case, in order to obtain scientifically accurate data, the patients that take the placebo are chosen randomly, and neither the doctors nor the patients know they are taking a placebo.**

23. In a clinical trial such as this, if you were assigned to the placebo group and took the placebo, following the conclusion of the clinical trial, would you want to be informed that the pharmaceutical you had taken was a placebo?

(a) Yes

(b) No **→ go to 25**

(c) Not sure **→ go to 25**

24. By which method would you prefer to be informed? (multiple answer allowed).

(a) In person

(b) By postal mail

(c) Over the phone

(d) By email or over a social network

(e) Not sure

25. Please state the reason.

[Fill in your answer: ]

**Currently, some feel that in order to prevent the development of unnecessary pharmaceutical products and to better understand the characteristics of the pharmaceutical products customers need, the process of developing pharmaceutical products should put greater emphasis on asking for patients’ opinions. In Europe and America, such efforts are in fact taking place, with people who have taken part in clinical trials contributing.**

26. Do you think greater emphasis should be put on asking for patients’ opinions on the following topics?

26-1. How participants in clinical trials want to be treated during the trial

(a) Patients’ opinions should be taken into account

(b) Patients’ opinions do not need to be taken into account

(c) Not sure

26-2. The suitability of explanatory/consent documents

(a) Patients’ opinions should be taken into account

(b) Patients’ opinions do not need to be taken into account

(c) Not sure

26-3. Which sorts of pharmaceutical products should be developed in the future

(a) Patients’ opinions should be taken into account

(b) Patients’ opinions do not need to be taken into account

(c) Not sure

26-4. Whether pharmaceutical product development plans are useful for patients

(a) Patients’ opinions should be taken into account

(b) Patients’ opinions do not need to be taken into account

(c) Not sure

26-5. Regarding decisions to continue or end development plans for particular pharmaceutical products

(a) Patients’ opinions should be taken into account

(b) Patients’ opinions do not need to be taken into account

(c) Not sure

27. Would you be able to state your opinions on the following?

27-1. How participants in clinical trials want to be treated during the trial

(a) I think I could state my opinion

(b) I don’t think I could state my opinion

(c) Not sure

27-2. The suitability of explanatory/consent documents

(a) I think I could state my opinion

(b) I don’t think I could state my opinion

(c) Not sure

27-3. The benefits of pharmaceutical products you are currently using

(a) I think I could state my opinion

(b) I don’t think I could state my opinion

(c) Not sure

27-4. The drawbacks of pharmaceutical products you are currently using

(a) I think I could state my opinion

(b) I don’t think I could state my opinion

(c) Not sure

27-5. Which sorts of pharmaceutical products should be developed in the future

(a) I think I could state my opinion

(b) I don’t think I could state my opinion

(c) Not sure

27-6. Whether a particular pharmaceutical product development plan would be useful for patients

(a) I think I could state my opinion

(b) I don’t think I could state my opinion

(c) Not sure

27-7. Whether the development plan of a particular pharmaceutical product should be continued or terminated, from a patient’s point of view

(a) I think I could state my opinion

(b) I don’t think I could state my opinion

(c) Not sure

28. Please state the reason.

[Fill in your answer: ]

29. Would you like to participate in a clinical trial in the future?

(a) I definitely would

(b) I suppose so

(c) Can’t say either way

(d) Not really

(e) Definitely not

**Finally, these questions will ask about you.**

30. Which one of the following descriptions applies to your physical condition over the past month or so? (Choose only one answer)

(a) Good

(b) A mix of good and bad

(c) Not good

31. What has your involvement with medical institutions been like over the past month or so? (Choose only one answer)

(a) I was just discharged from the hospital

(b) I receive outpatient treatment on a regular schedule

(c) I receive outpatient treatment as needed

(d) I do not receive outpatient treatment, but am under home care

(e) Other ( )

32. What is your age currently?

( ) years

33. What is your gender? (Choose only one answer)

(a) Male

(b) Female

34. Are you married? This includes common-law marriages. (Choose only one answer)

(a) I have a spouse

(b) I do not have a spouse (due to death)

(c) I do not have a spouse (due to divorce)

(d) I do not have a spouse (I have never been married)

35. What is your main occupation? (Choose only one answer)

(a) Student **→ go to 37**

(b) Company employee/company executive

(c) Civil servant/employee of non-government, non-corporate group

(d) Independent business/store owner **→ go to 37**

(e) Self-employed (doctor, lawyer, accountant, tax counselor) **→ go to 37**

(f) Other self-employed **→ go to 37**

(g) Contract employee, temporary employee **→ go to37**

(h) Part-time employee/freelance part-timer **→ go to 37**

(i) Other

(j) Not currently working **→ go to 37**

36. In which area do you work? (Choose only one answer)

(a) Farming, fishing, or forestry/mining/construction

(b) Manufacturing

(c) Electricity/gas/heat supply/water supply

(d) Telecommunications

(e) Shipping

(f) Trading/wholesale/retail

(g) Finance/insurance/real estate

(h) Service industry

(i) Medical care/welfare services

(j) Education/study support

(k) Other

37. How many people currently live in your household, including you? If someone is living away from the household temporarily, please do not include that person in your answer. If you live alone, please answer “1.” (Example: In a household with two parents and one child where the husband lives away from home for work, the answer would be “2.”)

( ) people

38. Please indicate the makeup of your household. (Choose only one answer)

(a) Single

(b) One generation

(c) Two generations

(d) Three or more generations

39. How many children do you have?

( ) children *If you do not have children, enter “0 (zero)”

40. How many of these children are under the age of 15?

41. Do you believe in a particular religion? (Choose only one answer)

* If you do not wish to answer, please choose “(g) Do not wish to answer.”

(a) Not religious

(b) Buddhist

(c) Christian

(d) Shinto

(e) Islam

(f) Other

(g) Do not wish to answer

42. What is the highest level of education you have completed? (Choose only one answer)

(a) Middle school

(b) High school (middle school under the former system)

(c) Vocational school

(d) Technical college/junior college

(e) University (high school under the former system)

(f) Graduate school

(g) Other

(h) Do not wish to answer

43. What was your approximate yearly household income last year? (Choose only one answer)

(a) Less than 3,000,000 yen

(b) 3,000,000-3,999,999 yen

(c) 4,000,000-5,999,999 yen

(d) 6,000,000-7,999,999 yen

(e) 8,000,000-9,999,999 yen

(f) 10,000,000-11,999,999 yen

(g) 12,000,000-14,999,999 yen

(h) 15,000,000-19,999,999 yen

(i) 20,000,000-29,999,999 yen

(j) 30,000,000 yen or more

(k) Do not know/do not wish to answer

44. Finally, if you have any requests or comments for health personnel involved in the clinical trial, or any comments regarding the questionnaire, please feel free to write them here.

( )
