# Supplementary material for: “Tell me what you suggest, and let’s do that, doctor”: Patient deliberation time during informal decision-making in clinical trials
Source: PLoS One. 2019 Jan 29;14(1):e0211338. doi: 10.1371/journal.pone.0211338 (PMC6350979; doi:10.1371/journal.pone.0211338)
Supplement: S1 Internet survey questionnaire in Japanese — (DOCX) [file pone.0211338.s002.docx]

**新しい薬や医療機器の開発に関わる治験への協力経験と意識に関する調査**

この調査は、新しい薬や医療機器の開発に関わる治験への協力経験と意識に関して、皆さまの考えや経験をおたずねするものです。結果は、新しい薬や医療機器の開発をよりよく進めるための体制づくりの資料とさせていただきます。調査の中で、もしお答えになりにくい場合は回答を中止していただいて結構です。また、ご協力は任意です。

　この調査は、**過去3年以内に治験に参加されたことのある患者さんを対象に**、日本医療研究開発機構からの助成を得て、東京大学が実施しています。

　この調査ではあなたのお名前や個人的な情報が公表されることはありません。また、結果を研究目的以外に使用することはありません。ご協力くださいますよう、どうぞよろしくお願いいたします。

以上についてお読みいただき、ご理解の上、調査にご協力いただける場合は、続いて質問への回答にお進みください。

**新しい医薬品を開発するときに、その安全性や効果を人で確認する研究を「臨床試験（りんしょうしけん）」または「治験（ちけん）」といいます。例えば、開発中の薬を飲んだり、機械を装着したり、手術や検査を受けたりしてその効果を調べる研究を実施します。**

**まず、「臨床試験」「治験」に関する以下の内容について、正しいと思うものをひとつ選んでください。**

1-1　「臨床試験」「治験」は、将来の医療の発展のために行われる。（○は１つだけ）

1. 正しい
2. 正しくない
3. 判断できない

1-2　「臨床試験」「治験」によっては、開発中の薬と比較するために薬としての成分を含まない安全な偽薬を飲むこともある。（○は１つだけ）

1. 正しい
2. 正しくない
3. 判断できない

1-3　「臨床試験」「治験」では、従来の治療を受けるか、それとも開発中の医療を受けるかを、患者は選べないことがある。（○は１つだけ）

1. 正しい
2. 正しくない
3. 判断できない

1-4　「臨床試験」「治験」には、病気の進行度によっては、協力できないことがある。（○は１つだけ）

1. 正しい
2. 正しくない
3. 判断できない

1-5　「臨床試験」「治験」を実施することによって、将来、標準的な治療が確立される。（○は１つだけ）

1. 正しい
2. 正しくない
3. 判断できない

1-6　「臨床試験」「治験」として行われる治療や薬の費用は、患者の負担ではない。（○は１つだけ）

1. 正しい
2. 正しくない
3. 判断できない

1-7　「臨床試験」「治験」に協力すると、患者の負担軽減のために謝礼が支払われる場合がある。（○は１つだけ）

1. 正しい
2. 正しくない
3. 判断できない

1-8　「臨床試験」「治験」に協力すると、一定の期間、定期的に受診したり、入院したりする必要がある。（○は１つだけ）

1. 正しい
2. 正しくない
3. 判断できない

1-9外国で標準的に使われている薬で、日本国内ではまだ承認されていないため、日本の患者がその薬を使えないことがある。

1. 正しい
2. 正しくない
3. 判断できない

1-10外国で標準的に使われている医療機器や検査で、日本国内ではまだ承認されていないため、日本の患者がその医療機器や検査を使った医療が受けられないことがある。

1. 正しい
2. 正しくない
3. 判断できない

**ここからの質問では、「治験」に関するあなたの体験談をうかがいます。**

**＊「治験」には、例えば、以下のようなものがあります。**

**・ 新薬を飲んで、その効果などを調べる研究**

**・ 日本で販売が承認されていない機械を装着して、その効果などを調べる研究**

2 治験に「協力した」または「協力を希望した」「協力を頼まれた」のは、いつごろでしたか。西暦または和暦でお答えください。複数のご経験がある方は、一番最近のものについてお答えください。

昭和（　　　　　　）年頃　または　平成（　　　　　　）年頃　または　西暦（　　　　　　　）年頃

3 その治験を知る最初のきっかけになったことを教えてください。

1. テレビの番組や広告を見て
2. 新聞・雑誌の記事や広告を見て
3. インターネットの記事や広告を見て
4. 病院内職員に誘われて
5. 病院内のポスターやパンフレットを見て
6. 家族や友人・知人に勧められて
7. 患者団体から情報を教えてもらって
8. 一般の人向けの講演会・セミナーで情報を知って
9. その他（具体的にお書きください：　　　　　　　）

4 治験に関する詳しい説明の書類（たとえば、「研究協力のお願い」など）を受け取ったことがありますか。

1. ある**→5へ**
2. ない**→　回答終了**
3. おぼえていない**→　回答終了**

**4で「治験に関する詳しい説明の書類（たとえば、「研究協力のお願い」など）を受け取ったことがある」とお答えになった方におたずねします**

5 その書類の内容について医師や看護師などから説明を受けましたか

1. 主に医師から受けた
2. 主に看護師から受けた
3. 主に「臨床試験（治験）コーディネーター」から受けた
4. 受けていない　**→16へ**
5. おぼえていない**→16へ**

5 あなたが受けた説明はわかりやすかったですか

1. 非常にわかりやすかった
2. ある程度わかりやすかった
3. あまりよくわからなかった
4. まったくわからなかった
5. おぼえていない

8 治験に協力することによる体によくないことや、副作用について説明を受けましたか

1. 受けた
2. 受けていない
3. おぼえていない

9 一度、治験に協力する同意をしたとしても、自由に協力をとりやめることができることについて説明を受けましたか

1. 受けた
2. 受けていない
3. おぼえていない

10 その書類を持ち帰って読みましたか

1. ひとりで読んだ
2. 家族や友人と読んだ
3. 読んでいない
4. おぼえていない

11 その書類は現在手元におもちですか

1. 保管している
2. 処分した
3. どこにしまったかわからない
4. おぼえていない

12 その治験に協力するかどうかについて、誰かと話し合ったり相談したりしましたか。（複数回答）

1. 医師や看護師など病院職員
2. 家族
3. 友人・知人
4. 同じ病気を持っている知人
5. 誰にも相談していない
6. その他（具体的にお書きください：　　　　　）

13 その治験に協力するかどうか、時間をかけて考えましたか。またその理由をお聞かせ下さい。

1. 非常に時間をかけてよく考えた【具体的にお書きください：　　　　】
2. ある程度時間をかけて考えた【具体的にお書きください：　　　　】
3. あまり時間をかけて考えなかった【具体的にお書きください：　　　　】
4. まったく時間をかけて考えなかった【具体的にお書きください：　　　　】
5. おぼえていない

14 治験への協力を決めた理由は何でしたか（複数回答）

1. 医療の進歩に貢献できると思ったから
2. 自分の病気の治療法の開発に貢献できると思ったから
3. これまでに自分が受けてきた医療に恩返しができると思ったから
4. 自分の病気が良くなるかもしれないと思ったから
5. 新しい治療（新薬を含む）を受けることができると思ったから
6. 自分の病気を治すには治験に協力するしかなかったから
7. 医療者からの説明に納得できた
8. 協力すると謝金（負担軽減費）がもらえるから
9. 医師に断りにくかったから
10. その他（具体的にお書きください：　　　　　　）

15 臨床試験や治験への協力を決めたときに、他の治療法を選ぶという選択肢もありましたか（複数回答）

1. 他の治療法がなかったので、治験を選んだ
2. 従来の治療法を選択することも可能だったが、治験を選んだ
3. 他に治療法があったのかどうか、わからない

**現在、治験が終了した後の患者さんとのコミュニケーションをもっとよくすべきではないかという意見があります。このことについて、あなたのお考えをうかがいます。**

16 いま、治験に協力したことについて、どのようにお考えですか

1. とてもよかった
2. まあよかった
3. ふつう
4. やや悪かった
5. とても悪かった

17 その理由をお聞かせください

【具体的にお書きください：　　　　】

18 あなたは今後、治験に協力したいと思いますか

1. そう思う
2. ややそう思う
3. どちらともいえない
4. あまりそう思わない
5. そう思わない

19 あなたが協力した治験の薬や医療機器などが、治験の終了後に、販売されたかどうかご存知ですか

1. 知っている**→20へ**
2. 知らない**→21へ**

20 どのようにして知りましたか

1. 医師から聞いた
2. 臨床試験（治験）コーディネーターから聞いた
3. 自分で調べた
4. その他（具体的にお書きください：　　　　）

21 あなたが協力した治験の薬や医療機器などが、治験の終了後に、販売されたかどうか知りたいですか

1. 知らせてほしい
2. 知らせてほしくない
3. 判断できない

22 知らせてもらう方法として、どのような手段を希望しますか。ただし、現在、製薬企業はあなたの個人情報を持っていません。あてはまるものにいくつでも○をしてください。

1. 病院から資料を郵送してほしい
2. 病院から電話をもらいたい
3. 病院でポスターを掲示したり、パンフレットを置いてほしい
4. 病院からメールやSNSで案内がほしい
5. 判断できない

**治験によっては、患者さんの協力を得て、薬としての成分を含まない安全な偽薬（プラセボ）を一定期間、服用していただくことにより、開発中の薬の効果と比較する場合があります。このような場合、科学的に正確なデータを得るため、偽薬を服用する患者さんを無作為に選び、医師や患者さんには偽薬を飲んでいることがわからないように実施されます。**

23 このような治験の場合、もしあなたが、偽薬を飲むグループに割り当てられ、偽薬を服用していたとしたら、治験の終了後に、あなたが飲んでいた薬が偽薬だったかどうか、知らせてほしいですか

1. 知らせてほしい
2. 知らせてほしくない**→25へ**
3. 判断できない**→25へ**

24 知らせてもらう方法として、どのような手段を希望しますか。あてはまるものにいくつでも○をしてください。

1. 対面で説明されたい
2. 郵便で教えてほしい
3. 電話で説明してほしい
4. メールやSNSで結果を送ってほしい
5. 判断できない

25 その理由をお聞かせください

【具体的にお書きください：　　　　】

**現在、無駄な医薬品の開発を防止したり、患者にとって必要な医薬品の性質をよく理解するため、患者からもっと参考意見を聞いて医薬品を開発すべきではないかという意見があります。欧州や米国では、実際にそのような取り組みを進めており、治験に参加した経験のある方の貢献が期待されています。**

26 あなたは、次のような場面で患者の意見をもっと聞くべきだと思いますか。

26-1 治験に参加する人が治験実施期間中にどのような配慮をしてほしいか

1. 患者の意見も反映されるべき
2. 患者の意見は反映されなくてよい
3. 判断できない

26-2 説明・同意文書の内容の適切さについて

1. 患者の意見も反映されるべき
2. 患者の意見は反映されなくてよい
3. 判断できない

26-3 今後どのような医薬品を開発すべきかどうか

1. 患者の意見も反映されるべき
2. 患者の意見は反映されなくてよい
3. 判断できない

26-4 ある医薬品の開発計画が、患者からみて有用そうかどうか

1. 患者の意見も反映されるべき
2. 患者の意見は反映されなくてよい
3. 判断できない

26-5 ある医薬品の開発計画の継続や終了についての判断について

1. 患者の意見も反映されるべき
2. 患者の意見は反映されなくてよい
3. 判断できない

27 あなたは、次のような意見を自分で述べることができそうですか。

27-1 治験に参加する人が治験実施期間中にどのような配慮をしてほしいか

1. 意見を言えると思う
2. 意見を言えそうにない
3. 判断できない

27-2 説明・同意文書の内容の適切さについて

1. 意見を言えると思う
2. 意見を言えそうにない
3. 判断できない

27-3 現在使用している医薬品の長所

1. 意見を言えると思う
2. 意見を言えそうにない
3. 判断できない

27-4 現在使用している医薬品の短所

1. 意見を言えると思う
2. 意見を言えそうにない
3. 判断できない

27-5 今後どのような医薬品を開発すべきか

1. 意見を言えると思う
2. 意見を言えそうにない
3. 判断できない

27-6 ある医薬品の開発計画が、患者からみて有用そうかどうか

1. 意見を言えると思う
2. 意見を言えそうにない
3. 判断できない

27-7 ある医薬品の開発計画について、患者からみて継続するか、それとも終了すべきか

1. 意見を言えると思う
2. 意見を言えそうにない
3. 判断できない

28 その理由をお聞かせください

【具体的にお書きください：　　　　】

29 あなたは今後、治験に協力したいと思いますか

1. そう思う
2. ややそう思う
3. どちらともいえない
4. あまりそう思わない
5. そう思わない

**最後に、あなたご自身のことについておたずねします。**

30 ここ1か月ほどの間のあなたのご体調は、以下のうちどれに当たりますか（○は１つだけ）

1. 良好である
2. 良好なときと、よくないときが混ざっている
3. よくない

31 ここ1か月ほどの間、あなたは医療機関にどのようにかかっていますか（○は１つだけ）

1. 退院したばかりである
2. 定期的に外来通院している
3. 自分が必要なときに外来通院している
4. 外来通院はせず、自宅療養中である
5. その他（　　　　　　）

32 あなたは現在何歳ですか。

（　　　　）歳

33 あなたの性別はどちらですか。（○は１つだけ）

1. 男性
2. 女性

34 あなたは結婚していますか。ここでの結婚には「事実婚」も含みます。（○は１つだけ）

1. 配偶者あり
2. 配偶者なし（死別）
3. 配偶者なし（離別）
4. 配偶者なし（婚姻歴なし）

35 あなたの主なご職業は何ですか。（○は１つだけ）

1. 学生**→37へ**
2. 会社員・会社役員
3. 公務員・団体職員
4. 自営業・商店主**→37へ**
5. 自由業（医師・弁護士・会計士・税理士）**→37へ**
6. その他自由業**→37へ**
7. 契約社員・派遣社員**→37へ**
8. パート・アルバイト・フリーター**→37へ**
9. その他
10. 現在仕事はしていない**→37へ**

36 あなたのお勤め先の業種は何ですか。（○は１つだけ）

1. 農林水産業・鉱業・建築業
2. 製造業
3. 電気・ガス・熱供給・水道業
4. 情報通信業
5. 運輸業
6. 商社・卸売・小売業
7. 金融・保険業・不動産業
8. サービス業
9. 医療・福祉
10. 教育・学習支援業
11. その他

37 現在あなたと一緒に住んでいる方は、あなたを含めて何人ですか。一時的に別居されている方がいる場合には、その方を含めずにお答えください。一人暮らしの方は「１人」とお書きください。

（例：夫婦と子ども1人の世帯で、夫が一時的に単身赴任の場合→2人）

（　　　　　　）人

38 あなたの世帯の構成を教えてください。（○は１つだけ）

1. 単身
2. 1世代
3. ２世代
4. ３世代以上

39 あなたのお子さまは、何人いますか？

（　　　　　　）人　※いない場合は「０（ゼロ）」と記入

40 そのうち15才未満のお子さまは、何人いますか

41 あなたは特定の宗教を信仰していますか（○は１つだけ）

　※回答することに抵抗がある方は、「キ．答えたくない」をお選びください

1. 無宗教
2. 仏教系
3. キリスト教系
4. 神道系
5. イスラム教系
6. その他
7. 答えたくない

42 あなたが最後に卒業された学校はどれですか。（○は１つだけ）

1. 中学校
2. 高校（旧制中学）
3. 専門学校
4. 高専・短大
5. 大学（旧制高校）
6. 大学院
7. その他
8. 答えたくない

43 あなたのお宅の昨年の収入（年収）は税込みでどれくらいでしたか。（○は１つだけ）

1. ３００万円未満
2. ３００～４００万円未満
3. ４００～６００万円未満
4. ６００～８００万円未満
5. ８００～１０００万円未満
6. １０００～１２００万円未満
7. １２００～１５００万円未満
8. １５００～２０００万円未満
9. ２０００～３０００万円未満
10. ３０００万円以上
11. わからない・答えたくない

44 最後に、治験に関わる医療者への要望・意見やこのアンケートの内容に関するご意見などがあればご自由にお書きください。

（　　　　　　　　　　　　　　　　　　　　　　　　　　　　　　　）
